# Ovotesticular Disorder of Sex Development: Approach and Management of an Index Case in the Dominican Republic

**DOI:** 10.7759/cureus.18512

**Published:** 2021-10-05

**Authors:** Manuel R De Jesus Escano, Miguel E Mejia Sang, Miguel Reyes-Mugica, Marc Colaco, Janelle Fox

**Affiliations:** 1 Urology, Memorial Sloan Kettering Cancer Center, New York, USA; 2 Department of Pediatrics, Lincoln Medical and Mental Health Center, Bronx, USA; 3 Department of Pathology, University of Pittsburgh Medical Center Children’s Hospital of Pittsburgh, Pittsburgh, USA; 4 Department of Urology, University of Pittsburgh Medical Center Children’s Hospital of Pittsburgh, Pittsburgh, USA

**Keywords:** genetics, sexual dysfunction, ambiguous genitalia, disorder of sexual development, ovotestis

## Abstract

Disorders of sex development (DSD) are a group of congenital conditions associated with anomalous development of internal and external genital organs. Ovotesticular disorder of sex development (OT-DSD) is a condition in which a child is born with both testicular tissue (that possesses variable fertility potential within seminiferous tubules) and ovarian tissue (with primordial follicles). These tissues may be co-existent in the same gonad (ovotestis) or independently in separate gonads.

Here, we report the clinical case of a 21-month-old boy that we met during a humanitarian surgical mission performed at Hospital Dr. Francisco Moscoso Puello, Santo Domingo, Dominican Republic. The child was referred for management of hypospadias, cryptorchidism, and symptomatic right inguinal and umbilical hernias. With further chromosomal evaluation, the diagnosis of SRY-negative OT-DSD was made, and shared decision-making was used to determine the timing of gender assignment, reconstruction, and the child’s long-term care team.

OT-DSD is an uncommon condition with unclear causes. Once a DSD condition is suspected at birth, a complete investigation should be performed, encompassing a descriptive examination, a basic electrolyte and hormonal profile, genetic assessment, and pelvic ultrasound. Consultation with a multidisciplinary team is warranted, including pediatric urology or pediatric surgery with urologic training, endocrinology, genetics, psychology, pathology, and the patient’s pediatrician at minimum before surgical reconstruction. It is crucial to involve the patient and their family with shared decision-making before surgery or gender assignment.

## Introduction

Disorders of sex development (DSD) are a group of congenital conditions associated with anomalous development of internal and external genital organs. These conditions are heterogeneous and should be further categorized as each condition has different fertility potential, heritability of a genetic mutation, potential for gender dysphoria and gonadal malignancy, and implications for gender assignment. Without classifying DSD, one can be easily overwhelmed by the heterogeneous presentation of these rare conditions. For example, some cases, including females with congenital adrenal hyperplasia and ovotestis DSD, are typically recognized at birth due to the ambiguity of the external genitalia. Other cases, such as pure gonadal dysgenesis or androgen receptor mutations, may present later in life with absent/delayed puberty or fertility issues [1]. Finally, an additional DSD condition, 5-alpha reductase deficiency, is known worldwide for small clusters of patients in the Dominican Republic. This condition presents with peripubertal virilization of phenotypic females who are actually genotypic males due to a mutation in the 5-alpha reductase gene. The two broad categories of DSD we must remember are disorders of gonadal development and disorders of androgen synthesis or action. Among the former, there are three subcategories: 46XX DSD, 46XY DSD, and sex chromosome DSD.

Ovotesticular disorder of sex development (OT-DSD), formerly called true hermaphroditism, is a condition in which a child is born with both testicular tissue (that possesses variable fertility potential within seminiferous tubules) and ovarian tissue (with primordial follicles). These tissues may co-exist in the same gonad (ovotestis) or independently (the ovary on one side and the testicle on the other) [2]. This is a rare condition as OT-DSD accounts for only 3-10% of DSD [2]. The global reported incidence of all DSD conditions is 1/5,500 newborns [3].

Here, we describe the clinical case of a classic OT-DSD patient who remained undiagnosed until he was referred to our service at nearly two years of age. A thorough clinical, genetic, and surgical approach was adopted to properly diagnose and manage the disorder.

## Case presentation

We present the case of a 21-month-old male patient seen during a humanitarian surgical mission performed at Hospital Docente Dr. Francisco Moscoso Puello, Santo Domingo, Dominican Republic. He was initially referred with the diagnoses of hypospadias, cryptorchidism, and umbilical hernia. He was born at 38 weeks to a young, healthy, and unrelated couple, and his medical history was unremarkable. Family history was negative for consanguinity, history of infertility, or history of genitourinary anomalies.

**Figure 1:**
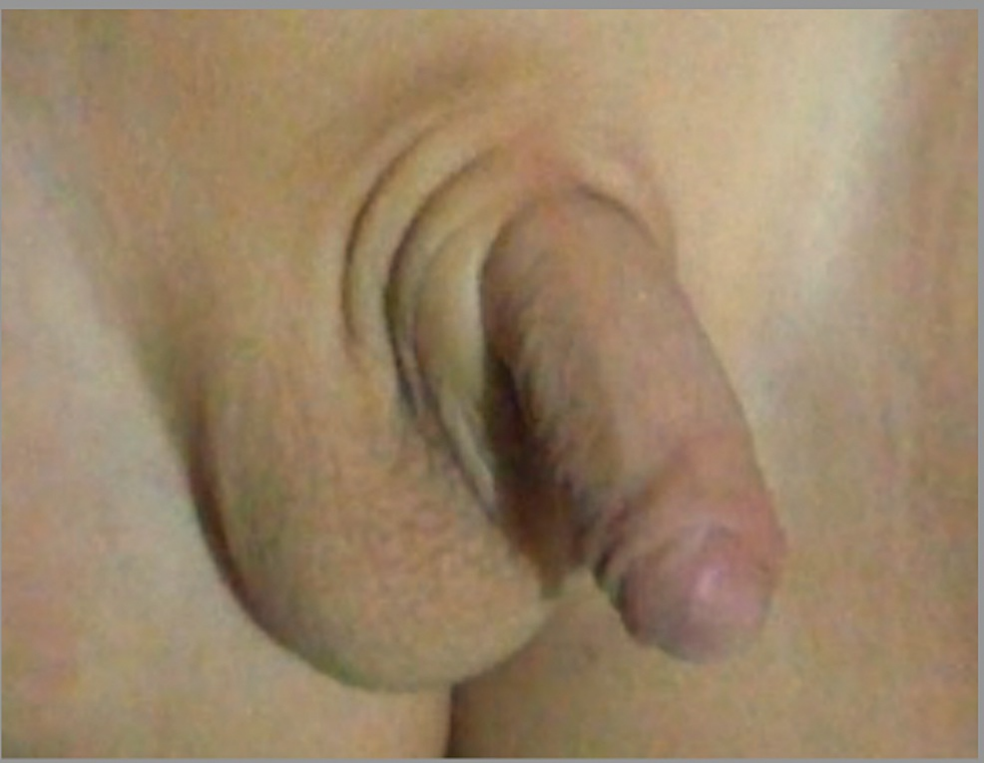
Ambiguous genitalia with Prader 4 phallus.

**Figure 2:**
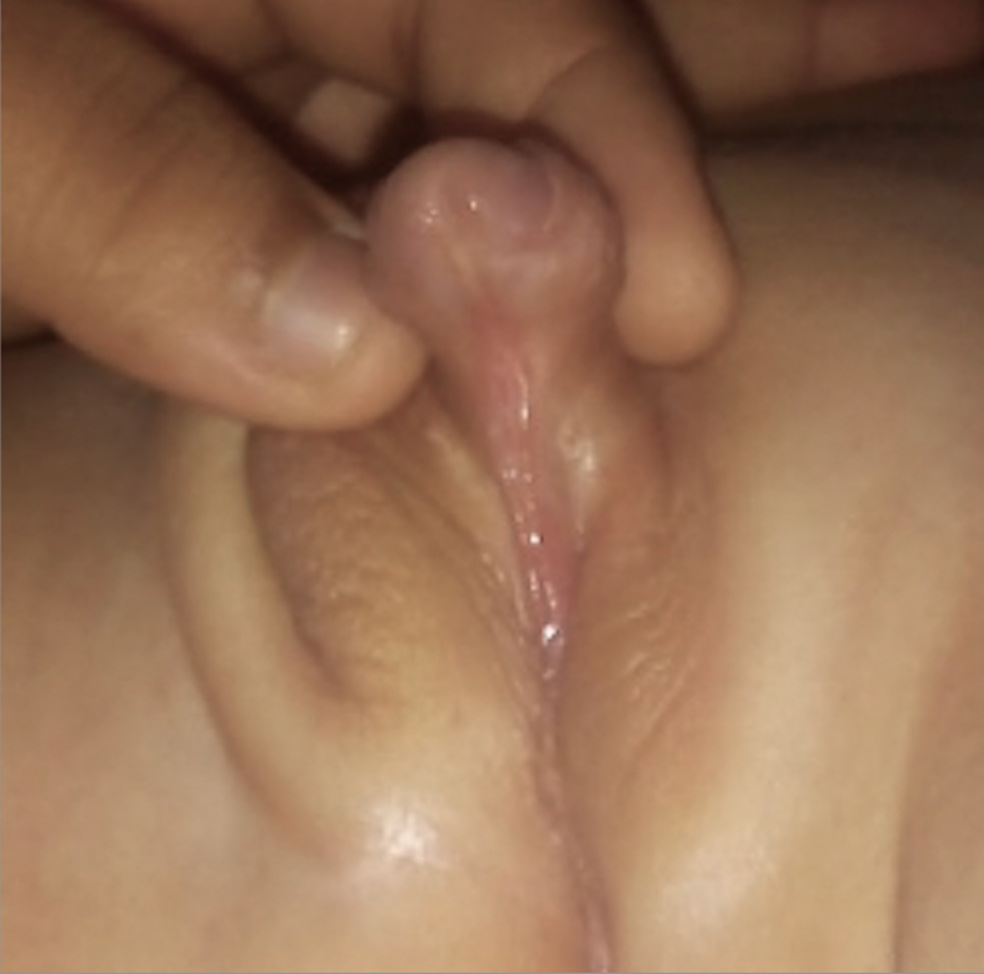
Single perineal opening.

**Figure 3:**
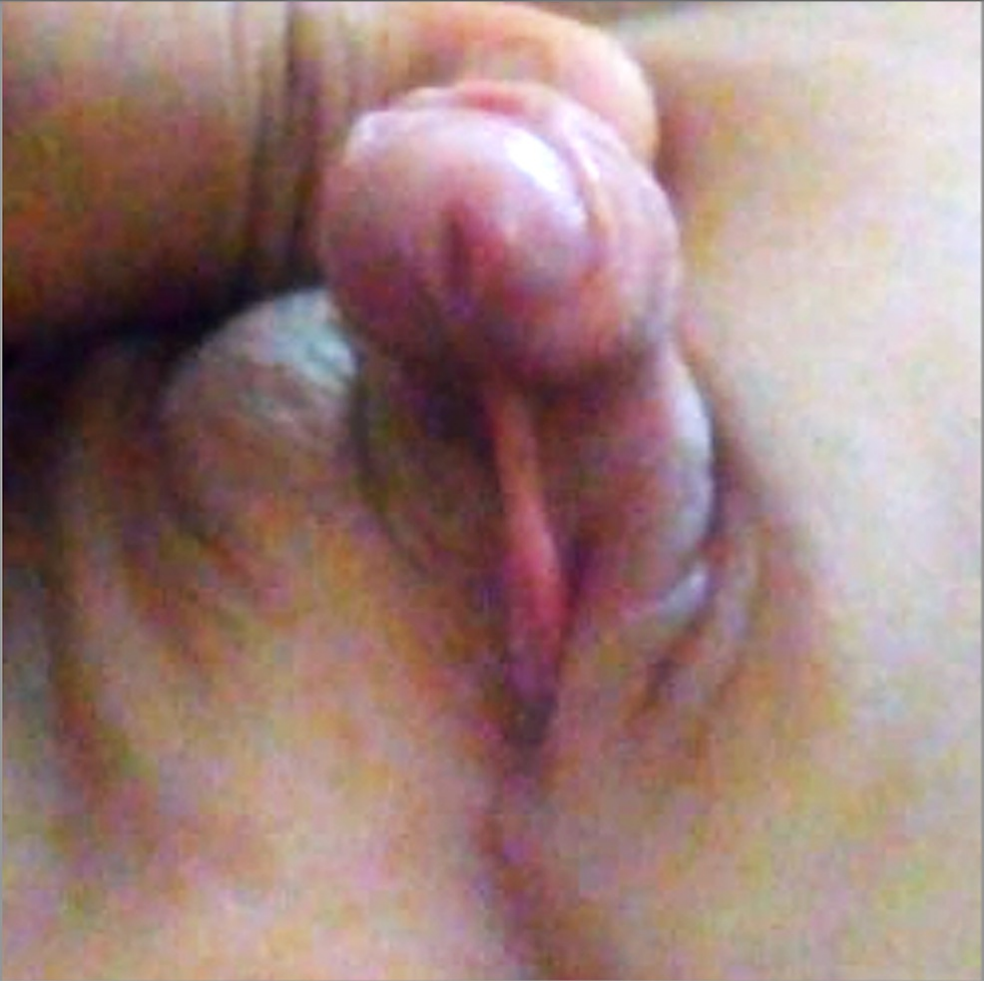
Bilateral nonpalpable testes.

**Figure 4:**
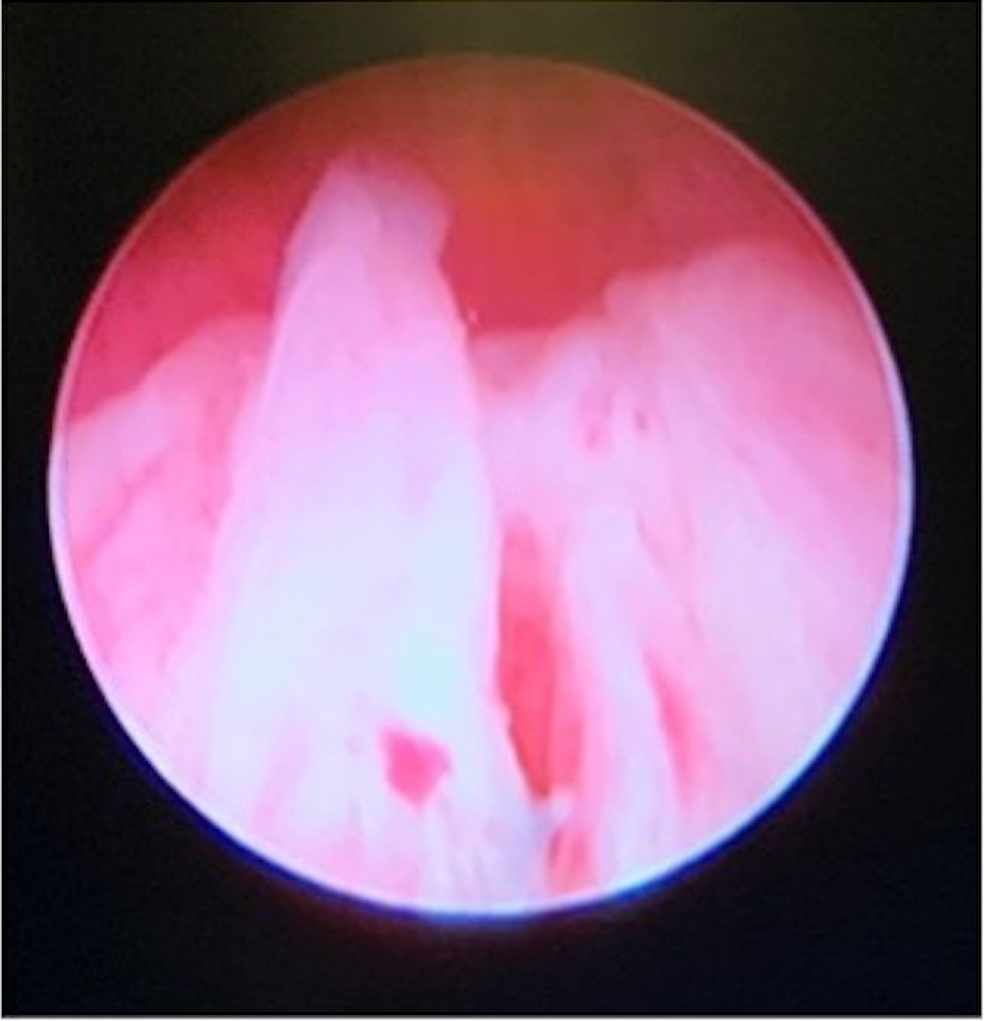
Cystoscopy findings showing an undervirilized prostatic urethra and the opening to a large utricle/Mullerian remnant.

**Figure 5:**
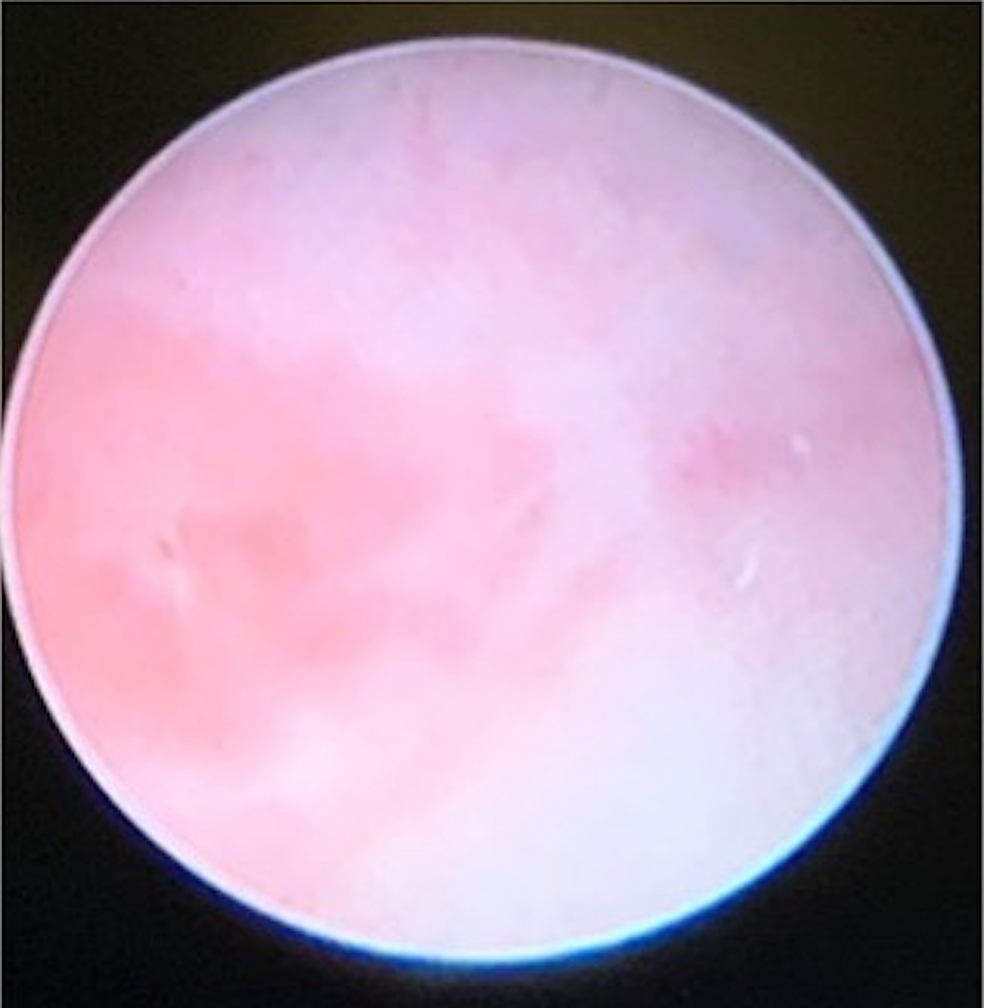
Cystoscopic findings: no cervix was visualized within the large utricle/Mullerian remnant, which connects with an atretic left-sided Mullerian remnant or uterine horn.

During the physical examination, we noticed ambiguous genitalia composed of a Prader 4 undervirilized phallus (Figure 1), perineal hypospadias (Figure 2), labioscrotal folds with nonpalpable testicles bilaterally (Figure 3), and a large symptomatic right inguinal hernia with an additional large umbilical hernia. A DSD condition was automatically in the differential due to nonpalpable testicles. The combination of undescended testes and proximal hypospadias further supported a diagnosis of DSD. Due to the symptomatic nature of the child’s hernias, the decision was made to proceed with right inguinal and umbilical hernia repairs as well as gonadal exploration with possible biopsy. A large bowel containing the right inguinal hernia was treated via high ligation, and the right gonad was inspected, revealing a suspected ovotestis on gross inspection. A vertical wedge biopsy was performed sampling both poles of the gonads and was sent both locally and to the University of Pittsburgh Medical Center (UPMC) Children’s Hospital of Pittsburgh for further evaluation with parental permission. Examination of the pelvis via the umbilical fascial defect demonstrated left-sided Mullerian remnants. Because a gonad was not readily visualized, the left groin was explored via a small inguinal incision and an ovary was identified. A cystoscopic evaluation was also performed noting bilateral ectopic ureters inserting near the neck bladder and a high confluence urogenital sinus 2 cm inside the meatus (Figure 4). Although no clear cervix was identified within the urogenital sinus, a small dimple was noted at the apex of the utricle or vaginal remnant, which may connect with an atretic uterine horn on the left (Figure 5).

**Figure 6:**
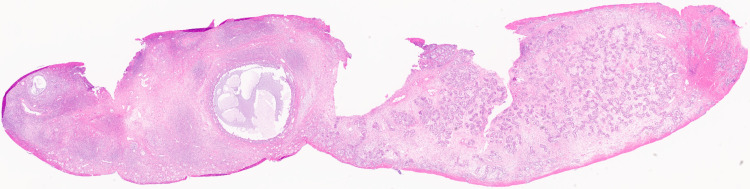
Low-power view of the bipolar ovotestis. The ovarian parenchyma is on the left, with follicles at different stages of development. On the right, there is testicular parenchyma with mildly dysgenetic testicular cords and expanded interstitial space (hematoxylin and eosin, 1×).

**Figure 7:**
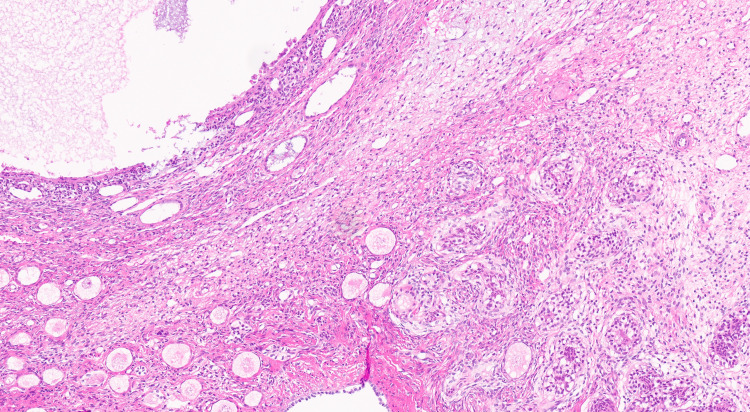
Area of the border between ovarian parenchyma (left) with multiple primary Graafian follicles and testicular parenchyma (right) with dysgenetic testicular cords (hematoxylin and eosin, 14×).

**Figure 8:**
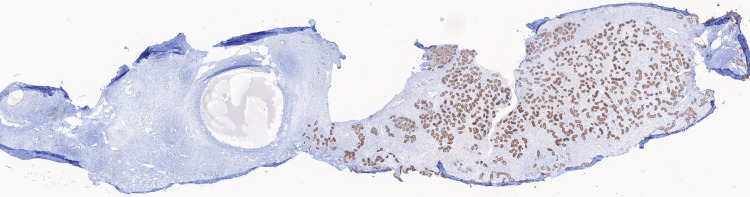
Immunohistochemical staining for inhibin is positive in Sertoli cells of testicular cords and completely negative in the ovarian parenchyma (left, 1×).

**Figure 9:**
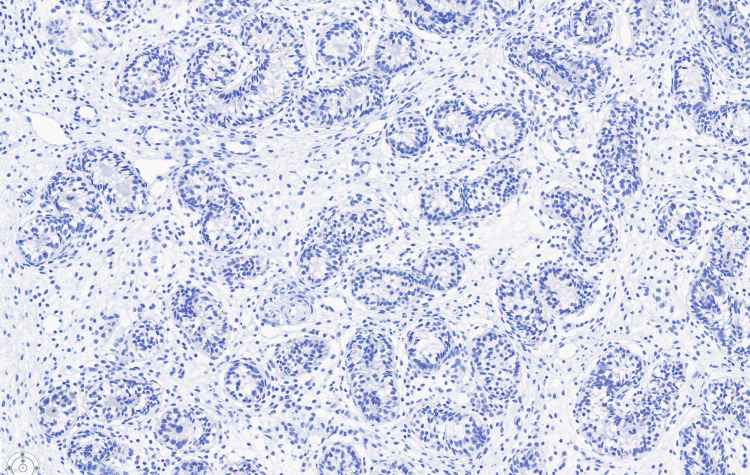
Immunohistochemical staining for SALL4, a marker of germ cells, is completely negative, indicating a fertility index of 0%. SALL4: Sal-like protein 4

Pathology from the right gonadal biopsy was reported by Hospital Docente Dr. Francisco Moscoso Puello as a bipolar ovotestis. Further characterization of the ovotestis was performed at UPMC Children’s Hospital of Pittsburgh noting a “boxcar appearance” or well-separated portions of the ovary and testicular parenchyma with approximately 50/50 distribution of each tissue (Figures 6-9). In addition to the normal ovarian stroma, there were seminiferous tubules with zero fertility potential. No gonadoblastoma or malignant tumors were noted. After surgery, chromosomal analyses were performed at UPMC Magee Women’s Hospital revealing 46XX chromosomes, negative fluorescence in situ hybridization (FISH) for the SRY gene, and negative X-chromosome microarray. An abdominal ultrasound was also unremarkable, and kidneys were normal.

After gathering all pertinent information, we consulted the parents’ preferences regarding gender assignment and their preferences for surgical timing. Shared decision-making was then used to discuss management options, including observation until the child could assign his or her gender, observation until parents were comfortable with a gender based upon child behavior, or surgeon recommendation based upon fertility and functional potential. A family practitioner, pediatrician, and surgeon met with the family virtually and elected to wait for further patient development and behavioral observation. The following year, the child’s family felt comfortable with male gender assignment and opted to proceed with surgical reconstruction prior to primary school matriculation. From a surgical standpoint, this will involve two to three stages of preputial and likely buccal graft urethroplasties to complete hypospadias repair, which will be combined with left oophorectomy, right partial gonadectomy to remove the ovarian remnant (preventing peripheral estrogen effects at puberty), and scrotoplasty. The risks of surgery cannot be understated and were discussed with the family, including roughly 50% chance of urethrocutaneous fistula or stricture repair, as well as additional risks of glans dehiscence, urethral diverticulum with post-void dribbling, persistent or recurrent penile curvature, and urinary tract infections (UTIs) or leakage related to the large utricle/Mullerian remnant. The latter should only be resected if it becomes symptomatic with recurrent UTIs. Given the risk of multiple surgical complications and the need for a multidisciplinary team, it was determined in the child’s best interest to refer to the Pediatric Surgery Department at the Hospital Infantil Dr. Robert Reid Cabral, which has the ability to coordinate referrals to psychology, endocrinology, genetics, and pediatric specialists.

## Discussion

DSD are a group of diseases characterized by abnormalities in the development of genes, gonad, or genital organs with different pathophysiological changes and clinical manifestations [4,5]. DSD are commonly identified in neonates with genital abnormalities or at adolescence with abnormal sexual development at the time of puberty. The management of DSD should be tailored to the specific condition and requires a multidisciplinary approach, including endocrinology, urology, radiology, genetics, psychology or child psychiatry, and the child’s pediatrician [6,7]. This is intended to optimize potential sexual function, genitourinary function, and address future issues of fertility, gender dysphoria, and long-term follow-up for potential surgical complications and functional outcomes [8]. Psychological assessment and counseling for both family and patient are critical when coming to a shared decision for treatment (sex assignment, hormonal therapy, and surgical intervention).

Initial DSD workup should include a detailed history and physical examination, electrolyte panel, hormonal panel, and imaging such as pelvic ultrasound to examine internal reproductive organs. For the examination, a description should be made of the external genitalia with Prader grading and the location/palpability of each gonad. For thorough hormonal evaluation, serum testosterone, dihydrotestosterone, estradiol, gonadotropins (luteinizing hormone, follicle-stimulating hormone), 17-hydroxyprogesterone, and androstenedione are crucial to identify the predominant sex cells. Additional labs may be helpful in a few circumstances. A normal male phenotype with nonpalpable testes, undetectable testosterone, high gonadotropins, and 46XY chromosomes can only be diagnosed with vanishing testes via undetectable inhibin-B and anti-Mullerian hormone, or diagnostic surgery such as laparoscopy. Plasma renin should be added upon the diagnosis of congenital adrenal hyperplasia. Lastly, it is crucial to obtain hormonal panels in the first few months of life as the gonads become quiescent after a period of infant puberty ending at approximately five months of age [9].

After initial workup, further investigation should include categorization of the DSD condition via genetic testing. First-line genetic investigation of an individual with DSD consists of chromosomal sex confirmation via karyotype, as well as identification of the Y-chromosome gene SRY via fluorescence in situ hybridization in 46XX virilized infants without congenital adrenal hyperplasia or in 46XY phenotypic females [10]. All neonates and young patients presenting with DSD also need comprehensive steroid and electrolyte analysis to avoid missing a potentially life-threatening acute adrenal insufficiency (e.g., congenital adrenal hyperplasia) [11]. Patients should also be screened for gonadal tumors as these can be found in at least 30% of individuals with XY gonadal dysgenesis or mixed gonadal dysgenesis, and are considered frequent (55%) in H-Y antigen-positive patients. These tumors begin almost always as precursor lesions termed gonadoblastomas in ovaries or dysgenetic gonads or germ cell neoplasia in situ (formerly known as intratubular germ cell neoplasia) in testes. As these lesions progress, they become dysgerminoma/seminoma (if the gonad is labeled as “ovary” or “testis,” respectively), both of which are malignant tumors. Complete androgen insensitivity may not have such a high risk as conditions with gonadal dysgenesis or dysplasia due to the low numbers of germ cells present in the gonads. Hence, some patients opt for orchiopexy of the gonads to palpable locations allowing progression through puberty naturally without estrogen supplementation [1]. Finally, testicular tumors of adrenal rests are possible in males with congenital adrenal hyperplasia [12]. The presence of Y chromosome material, including mosaic Turner syndrome (45XO with any percentage of Y chromosomes), warrants gonadectomy of all streak gonads and careful observation of the gonads left in situ [1]. In contrast, forms of gonadal dysgenesis not associated with Y chromosome material (e.g., 45,XO; 45,X/46,XX; 46,XX) have no definite increased risk for neoplasia.

OT-DSD patients with 46XX karyotype can have highly varied presentations; therefore, particular disorders may not be diagnosed based upon examination or phenotype alone. Gender assignment is also not as clear in this group of DSD patients as in congenital adrenal hyperplasia or complete androgen insensitivity. Fernández et al. described the case of a 13-year-old female patient with ambiguous genitalia who was not diagnosed with OT-DSD until a gonadectomy performed for torsion revealed both ovarian tissue and testicular tissue on pathological examination [7]. Roizza et al. described the case of a 27-year-old female 46XX/46XY mosaic patient with ambiguous genitalia and right ovotestis, who presented with delayed puberty as well as gender dysphoria. Unfortunately, the late presentation of this child with intra-abdominal gonads also resulted in delayed diagnosis of gonadoblastoma and mixed germ cell tumor [13]. Mendoza et al. described a 46XX infant with ambiguous genitalia not previously assigned a gender. After careful discussion, the female gender was elected by the family, and ovotestes were removed with the understanding that induction of puberty would be achieved with exogenous hormones only [14]. A final variation on the presentation of OT-DSD was described by Scarpa et al., who reported the case of a 46XY neonate with ambiguous genitalia and left ovotestis, with a uterus on ultrasound and very low serum testosterone. Hence, female sex assignment was suggested to the family [15]. In contrast, our patient demonstrated exclusively male behaviors, though his testosterone production will not be known until puberty, and he does not appear to have fertility potential in either gender. This is one of the most difficult situations in which we weigh the social implications of not assigning a gender or even delaying surgery until the child can choose, against the potential for gender dysphoria and patient dissatisfaction with their functional outcome if reconstructed at very young ages.

In the Dominican Republic, limited public providers with DSD expertise as well as the costs of out-of-pocket testing may hinder the early diagnosis of children with OT-DSD and other disorders of sex development. It is important to guide general pediatricians with a simplified means of categorizing DSD conditions and a concise workup that can be available in primary care settings. In our index case, the findings of ambiguous genitalia should have prompted at least chromosomal and hormonal workup prior to surgeon referral to predict testosterone production and brain imprinting in this child. The window for hormonal testing is lost after infant puberty at four to five months of age, and any attempts to stimulate the gonads with human chorionic gonadotropin can cause further virilization and at considerable expense of the test itself. Thus, after an infant is recognized to have ambiguous genitalia, we recommend the following laboratory evaluation at a minimum: basic metabolic panel to evaluate for salt-wasting (present in some children with congenital adrenal hyperplasia), 17-hydroxyprogesterone, testosterone, dihydrotestosterone, androstenedione, estradiol levels, and gonadotropins. Additionally, the patient should undergo a karyotype and pelvic ultrasonography to assess for Mullerian structures and ovaries. This information may then be used to categorize the child’s DSD into one of the following categories, which narrows the differential diagnosis: 46XX DSD, 46XY DSD, sex chromosome DSD, or 46XX or XY disorder of gonadal development (which includes OT-DSD). Syndromic presentations which include hypospadias or cryptorchidism and 46XY persistent Mullerian duct syndrome are separate categories as well [1]. Using this classification diagram may assist pediatricians with an earlier diagnosis and management by a multidisciplinary DSD team. Early diagnosis and less fragmented care may also prevent unnecessary surgery or inappropriate gender assignment with future gender dysphoria for the child [12].

## Conclusions

OT-DSD is an uncommon condition with unclear causes. Once a DSD condition is suspected, often due to ambiguous genitalia (hypospadias and one or more undescended gonads) at birth, a complete investigation should be performed, including a descriptive examination, a basic electrolyte and hormonal profile, genetic assessment, pelvic ultrasound, and consultation with a multidisciplinary team, including pediatric urology or pediatric surgery with urologic training, endocrinology, genetics, psychology, pathology, and the patient’s pediatrician at minimum before surgical reconstruction. It is crucial to involve the patient and the family with shared decision-making before surgery or gender assignment for avoiding gender dysphoria, respecting patient autonomy, and setting expectations on the goals of the reconstructive surgery as they pertain to future sexual function, voiding, fertility, and a host of reconstructive surgery complications. In short, the most crucial step is making an accurate initial diagnosis.
